# Effects of concentration, temperature and solvent composition on density and apparent molar volume of the binary mixtures of cationic-anionic surfactants in methanol–water mixed solvent media

**DOI:** 10.1186/2193-1801-2-280

**Published:** 2013-06-27

**Authors:** Ajaya Bhattarai, Sujeet Kumar Chatterjee, Tulasi Prasad Niraula

**Affiliations:** Department of Chemistry, M. M. A. M. C, Tribhuvan University, Biratnagar, Nepal

**Keywords:** Mixed solvent, Cetyltrimethylammonium bromide, Sodium dodecyl sulphate, Surfactant

## Abstract

The accurate measurements on density of the binary mixtures of cetyltrimethylammonium bromide and sodium dodecyl sulphate in pure water and in methanol(1) + water (2) mixed solvent media containing (0.10, 0.20, and 0.30) volume fractions of methanol at 308.15, 318.15, and 323.15 K are reported. The concentrations are varied from (0.03 to 0.12) mol.l^-1^ of sodium dodecyl sulphate in presence of ~ 5.0×10^-4^ mol.l^-1^ cetyltrimethylammonium bromide. The results showed almost increase in the densities with increasing surfactant mixture concentration, also the densities are found to decrease with increasing temperature over the entire concentration range, investigated in a given mixed solvent medium and these values are found to decrease with increasing methanol content in the solvent composition. The concentration dependence of the apparent molar volumes appear to be negligible over the entire concentration range, investigated in a given mixed solvent medium and the apparent molar volumes increase with increasing temperature and are found to decrease with increasing methanol content in the solvent composition.

## Introduction

The surfactants used in a multitude of industrial products, processes, and other practical applications almost always consist of a mixture of surfactants. Mixed surfactant systems are encountered in nearly all practical and industrial applications of surfactants. This is due to the natural poly-dispersity of commercial surfactants, which results from impurities in starting materials and variability in reaction products during their manufacture (Mata et al. 2004). Hence, one has the inherent difficulty preparing chemically and isomerically pure surfactants.

Mixed surfactant systems are much favored from the view-point of economy and performance. They are less expensive than isomerically pure surfactants and also they often provide better performance. Surfactant – surfactant interactions have been used extensively in industrial, pharmaceutical, technological, and biochemical fields. In the pharmaceutical field, for example, mixed micelle has been found to enhance the absorption of various drugs in human body (Aungst and Phang 1995; Tiwari and Saha 2013; Tengamnuay and Mitra 1990). A number of mixtures of cationic and anionic surfactant mixtures have been used in cleaning products to facilitate the dissolution and improved tolerance of water hardness (Ogino and Abe 1993). Due to their synergistic behavior at *cmc*, cosmetic industries use the mixed micelles in low concentrations to avoid potential skin irritation (Garcia et al. 1992; Robinson et al. 2005; Rhein et al. 1990). This synergistic phenomenon can also be highly beneficial for the environment as it allows the amount of surfactant released, and hence their impact, to be substantially reduced (Kibbey and Hayes 1997).

In view of the tremendous application potentials and economical consideration of a mixed micelle, it is necessary to search for the most suited surfactant combinations with desired requirements (such as, surface activity, solubility, catalytic property, etc.). In mixed micellar systems of ionic, nonionic and zwitterionic surfactants, three types of interactions may operate, *viz*., favorable (ionic-nonionic, ionic-zwitterionic and cationic-anionic), unfavorable and ideal mixing (nonionic mixtures).

Mixed surfactant systems are also of great theoretical interest. A mixed micellar solution is a representation of a mixed micelle, mixed monolayer at the air/solution interface, and mixed bilayer aggregate at the solid interface. In solutions containing two or more surfactants, the tendency of aggregated structures to form is substantially different from that in solutions having only pure surfactants. Such different tendency results in dramatic change in properties and behavior of mixed surfactants compared to that of single surfactant (Ogino and Abe 1993). Especially, mixing two surfactant ions of opposite charge, cationic/anionic surfactant mixtures show remarkably different physicochemical properties and behavior. For example, synergistic effects seem to be negligible for mixtures of nonionic surfactants. Ionic/nonionic mixtures, on the other hand, do show appreciable synergism (Jiang et al. 2009).

However, cationic/anionic surfactant mixtures exhibit the largest synergistic effects such as reductions in critical micelle concentration and surface tension (Menger and Shi 2009).

The basic idea is the hydrophobicity of the salts formed by the strong interactions between two different surfactants with opposite charge. To achieve better performance for detergent and cleaning product, mixed surfactants are commonly used to lower electrostatic forces between the surfactant heads. One of the best combinations to reduce such repulsive forces is by mixing anionic and cationic surfactants. The oppositely charged surfactants can act as counterions to each other and thus screen the repulsive forces (Sohrabi et al. 2008; Tondre and Caillet 2001; Li and Liu 1994).

As far as we know there is very little work in the literature dealing with the solution properties on binary mixtures of cetyltrimethylammonium bromide and sodium dodecyl sulphate (Tomasic et al. 1999) and no more work has been done on the effect of medium. In this paper, the results are reported for density measurements on sodium dodecyl sulphate in the presence of cetyltrimethylammonium bromide in methanol–water mixed solvent media with varying relative permittivity at different temperatures. Among various physical parameters, density and apparent molar volume have been recognized are the quantities that are sensitive to structural changes occurring in solutions (Hossain et al. 2010). The partial molar volume, *V*_A_ , is defined by Wandrey et al. (1999), as the following equation;1

where, ∂ *V* represent change in total volume and n as the number of moles. The partial molar volume is often provided in units of partial molar volume cm^3^/mol. If there is concentration dependence, the partial molar volumes have to be extrapolated to concentration zero using the following equation which calculates the apparent molar volume (*V*_B_) at the finite concentrations, c (Wandrey et al. 1999; De Lisi et al. 1990)2

where, M is the molecular weight of the sodium dodecyl sulphate, ***ρ***_0_ is the density of the solvent means the solution of cetyltrimethylammonium bromide in water and methanol–water mixed solvent media, ***ρ*** is the density of the solution and c is equivalent concentration in mol.l^-1^.

In order to calculate apparent molar volumes, the solution densities are measured for sodium dodecyl sulphate in presence of cetyltrimethylammonium bromide at the temperatures (308.15, 318.15, and 323.15) K in pure water and in methanol + water mixed solvent media containing (0.10, 0.20, and 0.30) volume fractions of methanol.

The aim of the present work is to analyze the influence of concentration, temperature and solvent composition of the binary mixtures of cationic-anionic surfactants in methanol–water mixed solvent media by densities measurement and the calculation of apparent molar volumes.

## Results and discussion

When methanol and water are mixed they occupy less volume than the sum of their volumes before mixing. In addition, the mixing can result in a temperature change, and also dissolved air may be eliminated. Measuring is made more complex by the fact that mixing methanol and water is exothermic. As a consequence the mixing effects are reduced in methanol, because the expansion due to heating compensates for the contraction due to mixing. The mixture was thoroughly shaken, and kept 24 hours for the released air bubbles to escape before attempting to make the solution of cetyltrimethylammonium bromide which was used to make the final solution of sodium dodecyl sulphate.

**Figure 1:**
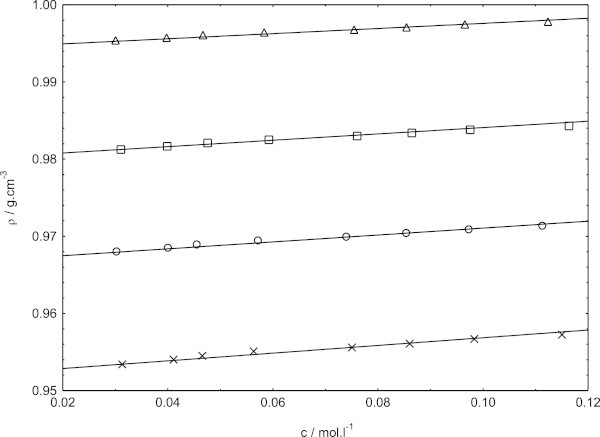
**Concentration dependence of density for sodium dodecyl sulphate in presence of cetyltrimethylammounium bromide at 308.15 K, in pure water (triangles) and different methanol–water mixtures (squares, in 0.1 volume fraction of methanol; circles, 0.2 volume fraction of methanol; crosses, 0.3 volume fraction of methanol).**

**Figure 2:**
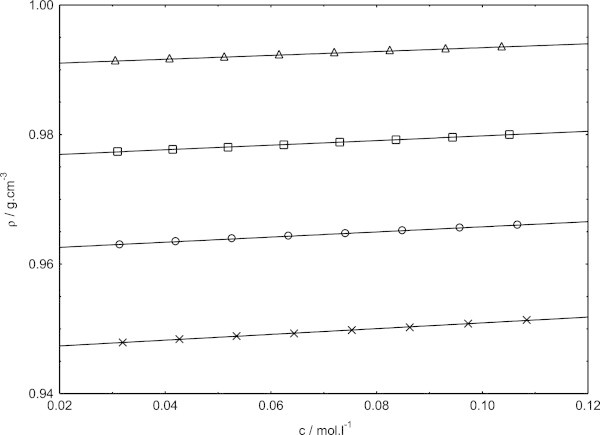
**Concentration dependence of density for sodium dodecyl sulphate in presence of cetyltrimethylammounium bromide at 318.15 K, in pure water (triangles) and different methanol–water mixtures (squares, in 0.1 volume fraction of methanol; circles, 0.2 volume fraction of methanol; crosses, 0.3 volume fraction of methanol).**

**Figure 3:**
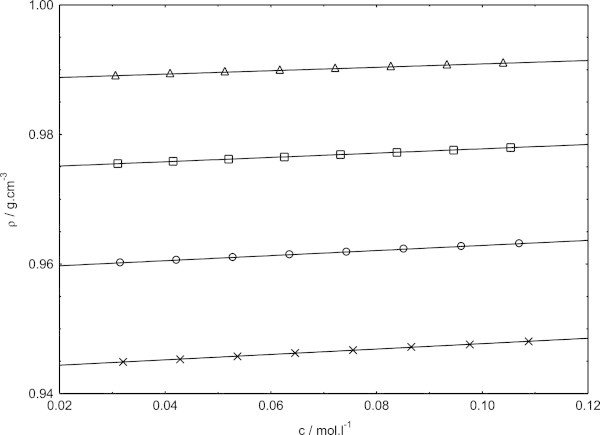
**Concentration dependence of density for sodium dodecyl sulphate in presence of cetyltrimethylammounium bromide at 323.15 K, in pure water (triangles) and different methanol–water mixtures (squares, in 0.1 volume fraction of methanol; circles, 0.2 volume fraction of methanol; crosses, 0.3 volume fraction of methanol).**

The densities for the sodium dodecyl sulphate in presence of cetyltrimethylammonium bromide in pure water and in three different methanol–water mixtures (containing 0.1, 0.2, and 0.3 volume fractions of methanol) at 308.15, 318.15, and 323.15 K are depicted in (Figures 1, 2 and 3)

Figures 1, 2 and 3 show the variation of densities of the investigated solutions as a function of the mixed surfactants concentration. From these figures, it is evident that the densities exhibits almost increase with increasing concentration within the concentration range investigated here. Our density data of sodium dodecyl sulphate in presence of cetyltrimethylammonium bromide in pure water and in methanol–water mixtures are found to be higher than the density data of sodium dodecyl sulphate in pure water and in methanol–water mixtures (Bhattarai et al. 2011). It is because the presence of cetyltrimethylammounium bromide with sodium dodecyl sulphate will naturally increase the density and decrease with increasing temperature (Chauhan et al. 2010). Obviously, the concentration dependence of density follows one and the same pattern at all the temperatures and solvent composition investigated. In fact, the variations of density with mixed surfactants concentrations are always found to be linear.

The slopes of density versus mixed surfactants concentration graph are always found to be positive in methanol–water mixtures, indicating strong ion-ion interactions in these media. The possible explanation for the positive slopes in the present mixed solvent media may be that the counterion binding would become quite appreciable in these media as the concentration of the surfactant is increased, thereby weaker ion-solvent interactions. As a consequence, contraction of the solvent would be gradually lowered with increasing concentration of the surfactant, resulting in a net positive volume change per mol of the added surfactant.

The effects of temperature and relative permittivity on the densities values are clear evident from (Figures 1, 2 and 3). At each temperature, the density values are found to decrease with decreasing relative permittivity in going from 0.1 volume fractions of methanol to 0.3 volume fractions of methanol over the entire concentration range investigated. The density is found to decrease in a given solvent media, with the increase in temperature as manifested in (Figures 1, 2 and 3). Evaluation of the solvent density lead to important insight as to the solution behavior of sodium dodecyl sulphate in presence of cetyltrimethylammonium bromide. The solvent density values thus obtained along with the slopes and the correlation coefficients of fits, (as r^2^ ) are listed in Table 1.

**Table 1:** **Density of the solvent (*ρ***_**0**_**), experimental slopes and the correlation coefficients of fits (as r**^**2**^**) of sodium dodecyl sulphate in presence of cetyltrimethylammonium bromide from Figures**1**,**2**and**3**in pure water and methanol–water mixtures at 308.15, 318.15 and 323.15 K**

	***T*** = 308.15 K			***T*** = 318.15 K			***T*** = 323.15 K		
Volume fraction of methanol	slope	***ρ***_0_	r^2^	slope	***ρ***_0_	r^2^	slope	***ρ***_0_	r^2^
0	0.033	0.9942	0.999	0.030	0.9904	0.999	0.026	0.9883	0.998
0.1	0.041	0.9799	0.999	0.036	0.9762	0.999	0.033	0.9745	0.999
0.2	0.045	0.9666	0.999	0.040	0.9618	0.998	0.039	0.9589	0.999
0.3	0.050	0.9519	0.998	0.044	0.9465	0.999	0.042	0.9435	0.999

Furthermore, at a given temperature, slopes are found to increase whereas the solvent density values are found to decrease as the solvent medium gets richer in methanol and the slopes as well as the solvent density values are found to decrease with increase of temperature in all compositions (Table 1). With increase in the methanol content, the relative permittivity of the medium decreases at a given temperature. Lower relative permittivity promotes greater counterion-binding and hence a lower amount of uncondensed counterions in going from 0.1 volume fractions of methanol to 0.3 volume fractions of methanol in the mixed solvent media over the entire range of temperatures investigated.

The review on catanionic systems between 1943 and early 1996 was done by Khan and Marques 1997 and they noticed that most of the mixed cationic-anionic surfactant systems, commonly known as catanionic systems, precipitate at equimolar concentrations at very high water content. At nonequimolar concentration, the mixture forms micelles with different sizes and shapes, closed bilayer vesicles and dilute lamellar phases. The formation of aggregates and their transformation are rationalized in terms of interaction forces and surfactant geometry. These features were also illustrated by Tomasic et al. (1999) on the mixed system of cetyltrimethylammonium bromide - sodium dodecyl sulphate. The precipitation occurred while mixing the equimolar amount of cationic (cetyltrimethylammonium bromide) and anionic (sodium dodecyl sulphate) surfactants. Therefore, while making the solution of surfactants’ mixture, we used excess amount of sodium dodecyl sulphate in comparison to cetyltrimethylammonium bromide because at a very high excess of one of the surfactants, the systems became clear due to mixed micelle formation (Tomasic et al. 1999).

The concentration of sodium dodecyl sulphate was taken more because sodium dodecyl sulphate interact with cetyltrimethylammonium bromide hydrophobically and favor micellization because anionic surfactants are known for having stronger hydrophobic interactions as compared with cationic surfactants (Bakshi, 1999a, 1999b).

**Figure 4:**
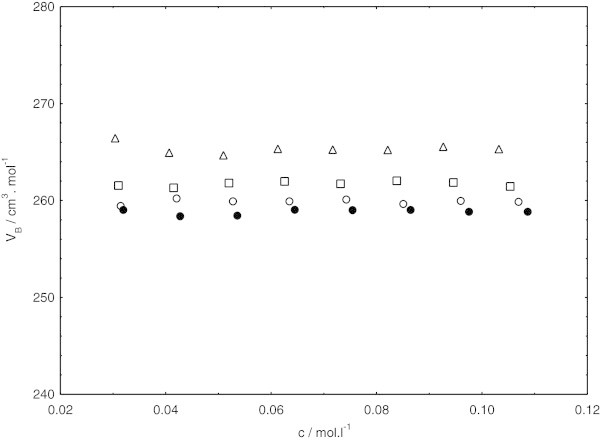
**Concentration independence of apparent molar volume for sodium dodecyl sulphate in presence of cetyltrimethylammounium bromide at 308.15 K, in pure water (triangles) and different methanol (1) + water (2) mixtures (squares, 0.10 methanol; circles, 0.20 methanol; closed circles, 0.30 methanol).**

**Figure 5:**
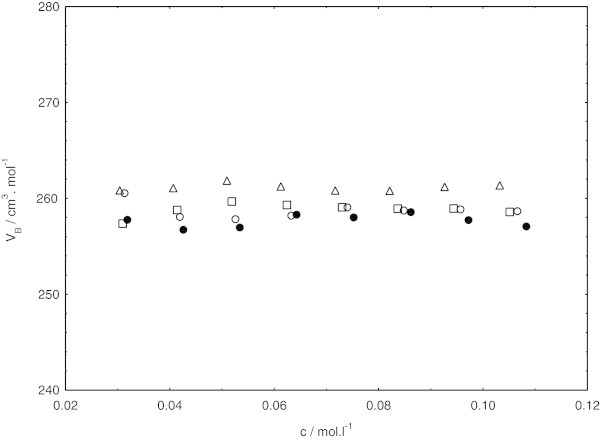
**Concentration independence of apparent molar volume for sodium dodecyl sulphate in presence of cetyltrimethylammounium bromide at 318.15 K, in pure water (triangles) and different methanol (1) + water (2) mixtures (squares, 0.10 methanol; circles, 0.20 methanol; closed circles, 0.30 methanol).**

**Figure 6:**
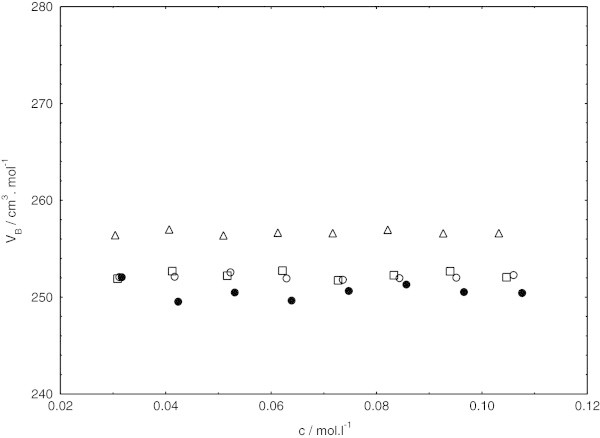
**Concentration independence of apparent molar volume for sodium dodecyl sulphate in presence of cetyltrimethylammounium bromide at 323.15 K, in pure water (triangles) and different methanol (1) + water (2) mixtures (squares, 0.10 methanol; circles, 0.20 methanol; closed circles, 0.30 methanol).**

The variation of apparent molar volumes for the sodium dodecyl sulphate in the presence of cetyltrimethylammonium bromide in pure water and three other different methanol + water mixtures containing (0.10, 0.20, and 0.30) volume fraction of methanol at (308.15, 318.15, and 323.15) K as a function of concentration are shown in Figures 4, 5 and 6.

From Figures 4, 5 and 6; the apparent molar volumes exhibit almost the same with increasing concentration within the examined concentration ranges in this study. Actually, in lower concentration of sodium dodecyl sulphate in the presence of cetyltrimethylammonium bromide, the apparent molar volumes were concentration dependent. Such behavior was also noticed by De Lisi et al. 1990 while calculating apparent molar volumes of alkyltrimethylammonium bromides. But the apparent molar volumes in lower concentration of sodium dodecyl sulphate in the presence of cetyltrimethylammonium bromide was not noted in our investigation because of irregular pattern of decrease in apparent molar volumes at low concentration of surfactants mixture.

In the examined concentration ranges, obviously, the concentration almost independence of apparent molar volumes follows the same pattern at all the temperatures and solvent compositions investigated. At each temperature, apparent molar volumes are found to decrease with decreasing relative permittivity by increasing the methanol content in the system. On the other hand, the apparent molar volume is increased in the given system with increasing temperature. This is mostly due to the weakening of surfactant-solvent binding energy with increasing temperature. The same pattern has been also reported by Iqbal and Siddiquah 2006.

## Conclusions

Experimental results for the density of salt-free solution of an anionic surfactant sodium dodecyl sulphate in presence of cationic surfactant cetyltrimethylammounium bromide in pure water and methanol–water mixed solvent media have been presented as a function of surfactant concentration and temperature. The densities are found to decrease with increasing temperature over the entire concentration range investigated in a given mixed solvent medium whereas these values are also found to decrease as the relative permittivity of the medium decreases. Estimation of the slopes and the calculated solvent density provide important insight regarding the solution behavior of mixed surfactants in methanol–water mixtures. With the help of density measurement, the calculated apparent molar volumes of sodium dodecyl sulphate in presence of cetyltrimethylammounium bromide have been presented as a function of surfactant concentration and temperature. The apparent molar volumes are found to increase with increasing temperature over the entire concentration range investigated in a given mixed solvent medium. Furthermore, at a particular temperature, the apparent molar volumes are found almost the same in the given concentration range of sodium dodecyl sulphate in presence of cetyltrimethylammounium bromide and these values are found to be decreased as the relative permittivity of the medium decreases.

## Methods

### Materials

Methanol (E. Merck, India, 99% pure) was distilled with phosphorous pentoxide and then redistilled over calcium hydride. The purified solvent had a density of 0.7772 g.cm^-3^ and a co-efficient of viscosity of 0.4742 mPa.s at 308.15 K; these values are in good agreement with the literature values (Apelblat 2011). Triply distilled water with a specific conductance less than 10^-6^ S.cm^-1^ at 308.15 K was used for the preparation of the mixed solvents. The physical properties of methanol–water mixed solvents used in this study at 308.15, 318.15, and 323.15 K are reported in Table 2 have been taken from the published papers(Bhattarai et al. 2011, 2006; Chatterjee and Das 2006). The relative permittivity of methanol–water mixtures at the experimental temperatures were obtained by regressing the relative permittivity data as function of solvent composition from the literatures (Albright and Gasting 1946; Yilmaz and Guler 1998) and are included in Table 2.

**Table 2:** **Properties of Methanol–water mixtures containing 0.10, 0.20 and 0.30 volume fractions of methanol at (308.15, 318.15 and 323.15) K**

T/***K***	***ρ***_0_/g.cm^-3^	***η***_0_/ mPa.s	D
	0.10 vol. fraction of methanol		
308.15	0.97973	0.8665	71.57
318.15	0.97604	0.7017	68.18
323.15	0.97438	0.6375	66.45
	0.20 vol. fraction of methanol		
308.15	0.96632	1.0217	68.14
318.15	0.96162	0.8075	64.80
323.15	0.95875	0.7300	63.15
	0.30 vol. fraction of methanol		
308.15	0.95160	1.1418	64.25
318.15	0.94626	0.8957	60.99
323.15	0.94331	0.8052	59.41

Cetyltrimethylammonium bromide was purchased from Loba Chemical Private Limited India and it was recrystallised several times until no minimum in the surface tension-concentration plot was observed and its critical micellar concentration (*cmc*) agreed with the literature value (Chakraborty et al. 2006).

Sodium dodecyl sulphate was purchased from Merck Specialities Private Limited India. It was recrystallised several times for purification. The minimum in the surface tension-concentration plot was observed. The aqueous solutions of purified and unpurified samples of sodium dodecyl sulphate exhibited minimum in the surface tension versus log *c* plot (*c*, concentration of sodium dodecyl sulphate). The minimum in the plot of *γ* versus log *c* for sodium dodecyl sulphate is considered as due to the presence of highly surface-active dodecyl alcohol molecules (Lin et al. 1999). Dodecyl alcohol may be present as impurity in the supplied sample of sodium dodecyl sulphate or it may be produced in the sodium dodecyl sulphate solution by its hydrolysis. The cmc of sodium dodecyl sulphate is taken to be the concentration of sodium dodecyl sulphate corresponding to the minimum in the plot of *γ* versus log *c* and it is equal to 8.10 mmol kg^−1^ in the absence of any added electrolyte at 25°C. This value is in good agreement with the cmc values of sodium dodecyl sulphate obtained from conductance (8.10 mmol kg^−1^) by Umlong and Ismail 2007.

### Apparatus and procedure

To measure density, the pycnometeric method was used. The stock solutions were freshly prepared for each concentration series to avoid problems of aging and microorganism contamination, which was found to occur with diluted surfactant solutions (No et al. 2000).

The densities of solutions were determined by the use of Ostwald-Sprengel type pycnometer of about 25 cm^3^ capacity. The sample solution was transfused into the pycnometer by using a medical syringe. The pycnometer was then tightly fixed in a thermostat at the experimental temperatures within ± 0.005 K. After thermal equilibrium was attained, the mass of the pycnometer was measured with electronic balance and the density was calculated. Density measurements are believed to be precise within ±0.00005, which is satisfactory for our purpose. In order to avoid moisture pickup, all solutions were prepared in a dehumidified room with utmost care. In all cases, the experiments were performed at least in three replicates.
